# Renal Cell Protection of Erythropoietin beyond Correcting The Anemia in Chronic Kidney Disease Patients

**Published:** 2013-11-20

**Authors:** Hamid Nasri

**Affiliations:** 1 Department of Nephrology, Division of Nephropathology, Isfahan University of Medical Sciences, Isfahan, Iran

**Keywords:** Erythropoietin, Erythropoiesis

## Abstract

Currently many patients with chronic renal failure have profited from the use of erythropoietin to correct anemia (1,2). In chronic kidney disease, anemia is believed to be a surrogate index for tissue hypoxia that continues preexisting renal tissue injury (1-3). Erythropoietin is an essential glycoprotein that accelerates red blood cell maturation from erythroid progenitors and facilitates erythropoiesis. It is a 30.4 kD glycoprotein and class I cytokine containing 165 amino acids (3,4). Approximately 90% of systemic erythropoietin in adults is produced by peritubular interstitial fibroblasts in the renal cortex and outer medulla of the kidney (3-5). A feedback mechanism involving oxygen delivery to the tissues seems to regulate erythropoietin production. Hypoxia-inducible factor regulates transcription of the erythropoietin gene in the kidney, which determines erythropoietin synthesis (3-5). Erythropoietin is an essential glycoprotein that accelerates red blood cell maturation from erythroid progenitors and mediates erythropoiesis in the bone marrow (4-6). Kidney fibrosis is the last common pathway in chronic renal failure irrespective of the initial etiology (5,6). Constant inflammatory cell infiltration and pericyte-myofibroblast transition lead to renal fibrosis and insufficiency which result in decreased production of erythropoietin (4-7). Thus far, therapeutic efforts to treat patients with chronic renal failure by administering erythropoietin have been made only to correct anemia and putative hypoxic tissue damage. The introduction of recombinant human erythropoietin has marked a significant advance in the management of anemia associated with chronic renal failure (6-9). With an increasing number of patients with chronic renal failure receiving erythropoietin treatment, emerging evidence suggests that erythropoietin not only has an erythropoietic function, but also has renoprotective potential. In fact, in recent years, the additional non-erythropoietic tissue/ organ protective efficacy of erythropoietin has become evident, especially in the kidneys (5-12). Various investigations have shown the kidney protective property of erythropoietin in acute kidney injury. In a study to evaluate the ameliorative effects of erythropoietin on renal tubular cells, we studied 40 male rats. We found that erythropoietin was able to prevent the increase in serum creatinine and blood urea nitrogen. Furthermore, co-administration of gentamicin and erythropoietin effectively reduced kidney tissue damage compared to the control group. However, the protective properties of erythropoietin were also evident in our study. When the drug was applied after gentamicin- induced tubular damage we were able to show that the drug was still effective after tissue injury onset. This indicates that erythropoietin may have curative effects in addition to its preventive properties (13). Thus, erythropoietin is a promising kidney protective agent to prevent, ameliorate or attenuate tubular damage induced by gentamicin or other nephrotoxic agents that act in a similar manner to this drug (14-17). Recent studies have elucidated the cellular mechanism involved in kidney erythropoietin production and the consequent events that lead to kidney fibrosis, showing that they are closely related to each other (18-20). In contrast to previous findings, fibroblasts originating from damaged renal tubular epithelial cells do not have an important role in kidney fibrosis, but renal erythropoietin- producing cells, stemming from neural crests, have been shown to trans-differentiate into myofibroblasts after long-term exposure to inflammatory situations related to kidney fibrosis. In fact, almost all myofibroblasts expressing α-smooth muscle actin originate from renal erythropoietin-producing cells, which are naturally peritubular interstitial fibroblastic cells expressing neural cell marker genes but not α-smooth muscle actin. Macrophages and myofibroblasts are responsible for fibrosis in the renal tissue. Macrophages could be differentiated to phenotype M1 (classically activated) or M2 (wound healing) according to the distinctive cytokine production and behavior that follows different routes of activation (6,8,21,22). While erythropoietin can disengage macrophages by stopping the activity of NF-κB, it is possible that one of the mechanisms explaining the antifibrotic effects of erythropoietin in chronic kidney disease is *in vivo* macrophage regulation (20-25). These important findings stipulate the missing link in chronic renal failure between anemia and kidney fibrosis (6,8,21,22). In patients with chronic kidney disease, anemia due to reduced erythropoietin production eventually appears (1,4,5). Recombinant human erythropoietin has been used for more than 20 years in chronic kidney disease to recompense for reduced endogenous erythropoietin production (1,4,5,25). Recent investigations have pointed out that erythropoietin administration improves kidney functions in chronic kidney disease either directly or indirectly (17-24). The therapeutic benefits of erythropoietin beyond the correction of anemia are still questioned. However, it is notable that various pieces of evidence simply reflect the pleiotropic effects of erythropoietinon on the central nervous, cardiovascular system and on the kidney (18,20,25). In brief, clinical evidence shows the kidney protective potential of erythropoietin in patients with chronic renal failure, however, additional clinical investigations are crucial to outline when to start erythropoietin treatment and what is the optimal erythropoietin dosage for slowing disease progression in patients with chronic renal failure. The application of erythropoietin treatment for renoprotection may need to be earlier than that for erythropoiesis, while it is possible that the erythropoietin attenuation of renal fibrosis through macrophage regulation and endothelial cell protection operates through other unidentified mechanisms. While agents restoring the initial function of renal erythropoietin-producing cells could delay kidney fibrosis, further laboratory studies are necessary to clarify the cellular target of erythropoietin in the kidney and for developing a novel erythropoietin derivative or mimetic for kidney protection.
